# Quick and Label-Free Detection for Coumaphos by Using Surface Plasmon Resonance Biochip

**DOI:** 10.1371/journal.pone.0104689

**Published:** 2014-08-14

**Authors:** Ying Li, Xiao Ma, Minglu Zhao, Pan Qi, Jingang Zhong

**Affiliations:** 1 Key Laboratory of Optoelectronic Information and Sensing Technologies of Guangdong Higher Education Institutes, Jinan University, Guangzhou, Guangdong, China; 2 Pre-university Department, Jinan University, Guangzhou, Guangdong, China; 3 Department of Optoelectronic Engineering, Jinan University, Guangzhou, Guangdong, China; 4 Department of Electronics Engineering, Guangdong Communication Polytechnic, Guangzhou, Guangdong, China; CNR, Italy

## Abstract

Coumaphos is a common organophosphorus pesticide used in agricultural products. It is harmful to human health and has a strictly stipulated maximum residue limit (MRL) on fruits and vegetables. Currently existing methods for detection are complex in execution, require expensive tools and are time consuming and labor intensive. The surface plasmon resonance method has been widely used in biomedicine and many other fields. This study discusses a detection method based on surface plasmon resonance in organophosphorus pesticide residues. As an alternative solution, this study proposes a method to detect Coumaphos. The method, which is based on surface plasmon resonance (SPR) and immune reaction, belongs to the suppression method. A group of samples of Coumaphos was detected by this method. The concentrations of Coumaphos in the samples were 0 µg/L, 50 µg/L, 100 µg/L, 300 µg/L, 500 µg/L, 1000 µg/L, 3000 µg/L and 5000 µg/L, respectively. Through detecting a group of samples, the process of kinetic reactions was analyzed and the corresponding standard curve was obtained. The sensibility is less than 25 µg/L, conforming to the standard of the MRL of Coumaphos stipulated by China. This method is label-free, using an unpurified single antibody only and can continuously test at least 80 groups of samples continuously. It has high sensitivity and specificity. The required equipments are simple, environmental friendly and easy to control. So this method is promised for a large number of samples quick detection on spot and for application prospects.

## Introduction

Organophosphorus pesticide (OPPs) is a type of phosphate with different substituent groups that cause the inhibition of acetylcholinesterase to produce an insecticidal effect [1]–[3]. China is one of the world’s leading users of pesticide, nearly 70% of which is organophosphorus. Organophosphorus pesticide often has high levels of neurotoxicity in human and animals through gradual accumulation and regular intake. Organophosphorus pesticide may lead to symptoms such as neurological disorders, tremors, language disorders and even death [4]–[15]. Due to its high levels of toxicity, many organophosphorus pesticides have been banned or highly limited in use by most countries. Efforts are being made to revamp restrictions and regulations of organophosphorus pesticide residues in imported fruits and vegetables across America, Europe and Asia, which makes improved detection methods urgent and necessary.

Coumaphos is one of the organophosphorus pesticides. Coumaphos is harmful to human health and has a strictly stipulated maximum residue limit (MRL) on fruits and vegetables. China stipulates that the residues of Coumaphos in vegetables and fruits must be lower than 0.05 mg/kg [16]. Currently, it can be detected through methods of chromatography such as low pressure gas chromatography-mass spectroscopy (LP GC-MS) [17]–[20], high performance liquid chromatography (HPLC) [21]–[23] and thin layer chromatography (TLC) [24]–[25]. Additionally the wave spectrum method [26]–[28] and enzyme-linked immunosorbent assay (ELISA) [29]–[30], are also used.

Although these methods are highly sensitive and accurate, the sample pretreatment procedures are time-consuming and labor intensive, complex in execution and require expensive equipment. Furthermore, they do not enable real-time detection and rapid screening of large amount of samples. In contrast, the surface plasmon resonance biochip method is quick, highly sensitive, and label-free, and has been widely used in pharmaceutical analysis, food analysis, environment monitoring and many other fields [31]–[33]. This study discusses a detection technique based on surface plasmon resonance in organophosphorus pesticide residues, for example Coumaphos. This research adopts the self-developed portable surface plasmon resonance biochip detector by using of the specificity of immunoreaction to study the detection of higher toxicity organophosphorus pesticide-Coumaphos, to analyze the process of kinetic reactions and to establish its standard curve. The technique, which is based on surface plasmon resonance (SPR) and immune reaction, belongs to the suppression method. Comparing with ELISA methods and other methods, this method is label-free and has high specificity, and the sample pretreatment procedure is simple. The utilized equipment is cheap, simple, environmental friendly and easy to control. It achieves the continuousdetection and the quick screening of a large number of samples. This method can be applied in places where real-time quick detection and quality control is needed such as supermarkets, bazaars and factories.

## Materials and Methods

### Instrument and equipment, materials and reagents

The self-constructed portable SPR biochip detector is a sensitive and accurate angle scanning device. The use of this device is simple and easy, with a precision of up to 0.002°. It can also be controlled easily by LabView and has a user friendly interface [34].

Coumaphos standard (0.02 g/mL, the molecular weight of 362.78) is obtained from Dr. Ehrenstorfer GmbH (Augsburg, Germany). H_11_-OVA (Coumaphos-ovalbumin, 83 mg/L) and unpurified monoclonal antibody ascites of Coumaphos (5 mg/mL) are produced by the College of Food Science, South China Agricultural University, as described in the previous work [35]–[36]. HS(CH_2_)_10_COOH (mercapto-undecanoic acid) and HS(CH_2_)_6_OH (mercapto-hexanoate), N-hydroxysuccinimide (NHS), N-ethyl-N’-(dimethylaminopropyl) carbodiimide (EDC), Ethanolamine (Eth), and Sodium dodecyl sulfate (SDS) are purchased from Sigma, USA. Other reagents are purchased from Beijing Chemical Reagent Company. The PBS buffer (2 mmol/L NaH_2_PO_4_, 2 mmol/L Na_2_HPO_4_, 150 mmol/L NaCl, pH 7.4) is used as immune reaction buffer. Coumaphos antigen and monoclonal antibody ascites of Coumaphos are diluted to the appropriate concentration solution with PBS buffer.

### Preparation of Biochip

**Figure 1:**
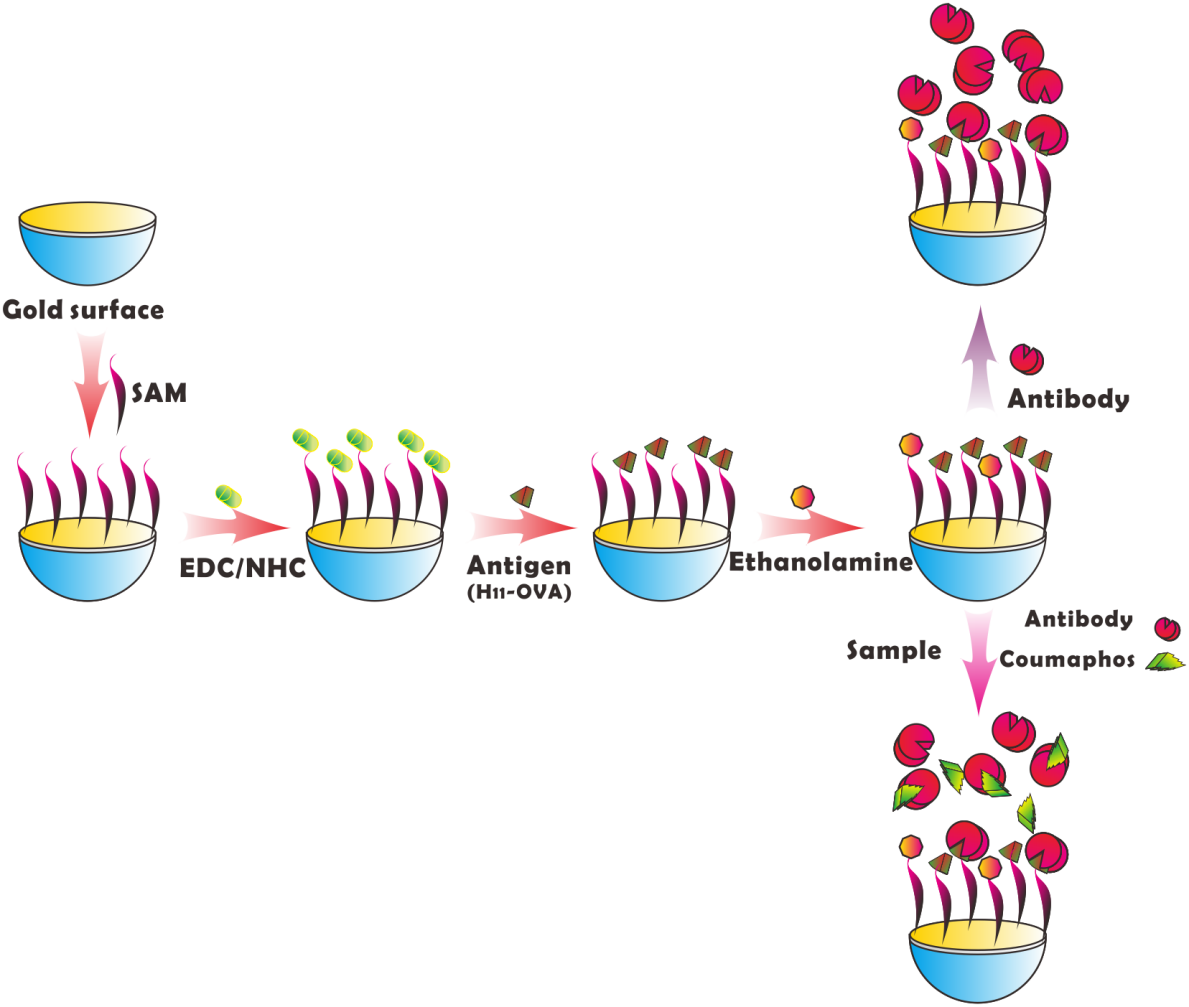
Scheme of Coumaphos immune detection by surface plasmon resonance biochip.

A gold film (thickness of 50 nm) is deposited on the surface of a piece of glass (diameter of 20 mm and thickness of 1 mm). The biochip with gold film is attached to the instrument. The flow cell is installed, and the PBS buffer is injected into it. The biochip will be self-assembledwhen the baseline is stable for a few minutes. The ethanol solution of mercaptoundecanoic acid (0.1 mmol/L HS(CH_2_)_10_COOH and 0.9 mmol/L HS(CH_2_)_6_OH (Mercaptohexyl hexanoate)) are injected into the flow cell. The gold film is chemically modified with self-assembled monolayers (SAMs) by the prepared solution for 2 hours. The sulfhydryl terminal of the modified liquid can interact with gold and they bond to each other (S-Au bond), and then adsorb on the gold film stably and orderly. C-terminus can be activated to active ester by EDC/NHS as active groups. Active ester can react with proteins (antibody or antigen) to form amide linkage which will fix protein. After the biochip is modified, the PBS buffer is injected to wash the biochip for forming the baseline of the whole response. The 0.1 mol/L NHS and 0.1 mol/L EDC (1∶1, V/V) are added on to the surface of the biochip after the baseline being stable to activate the biochip for 15 minutes. The bioprobe H_11_-OVA is fixed after washed by PBS for 2 minutes. The H_11_-OVA which has been diluted 15 times as bioprobe is fixed on the surface of the biochip. The response value of the SPR increases significantly. Thirty minutes later, the biochip is washed by PBS for two minutes. The response value of the SPR after fixing is higher than it was before fixing, meaning that the effect of the fixed bioprobe is better. The ethanolamine (pH 8.5, 1 mol/L) is added for the inactivation of the remaining ester bond. The PBS is used to wash the gold film 5–7 minutes after for inactivation. The preparation of the biochip is complete and it can be used for the following detection. The process of Coumaphos immune detection by surface plasmon resonance biochip is shown in Fig. 1.

### Suppression Detection

If the probe of biochip is the antibody of Coumaphos, Coumaphos, with a molecular weight of 362.78, can be combined with the antibody that is fixed on the surface of the biochip. This is the direct detection of Coumaphos, but the effect of the refractive index changing around the biochip surface is small. The effect of the direct detection will be unsatisfied because the variation of resonance peak is small. For the trace detection of substances with small molecular weight like Coumaphos, the suppression method was proposed. The bioprobe of the biochip is H_11_-OVA. 5 minutes after the antibodies were mixed with different concentrations of Coumaphos, the resultant mixtures are added on the surface of the biochip. The SPR effect is detected and the kinetic process can be analyzed. If the concentration of Coumaphos molecules is low, more antibodies of Coumaphos will combine with the probes H_11_-OVA on the surface of the biochip, and then the response value of SPR will become larger. The concentration of Coumaphos molecules is inversely proportional to the response value of SPR. That is the meaning of the method of suppression detection.

## Results and Discussion

**Figure 2:**
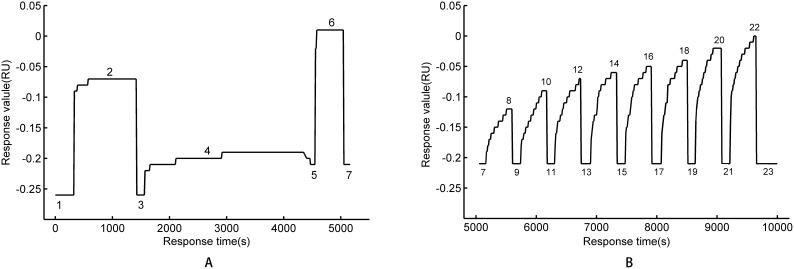
The process of a group of Coumaphos molecules detected by suppression method. A) The preparation of the biochip. 1: PBS; 2: active ester; 3: PBS; 4: The fixation of the bioprobe H_11_-OVA; 5: PBS; 6: inactivated; 7: PBS. B) Detecting a group of samples by suppression method. 8, 10, 12, 14, 16, 18, 20, 22 plot samples 5000 µg/L, 3000 µg/L, 1000 µg/L, 500 µg/L, 300 µg/L, 100 µg/L, 50 µg/L and 0 µg/L, respectively. 7, 9, 11, 13, 15, 17, 19, 21, 23 are PBS.

The process of a group of Coumaphos molecules detected by suppression method is shown in Fig. 2. It plots the baseline of PBS in Marking 1 in Figure 2A, the activation of the surface of the biochip, and c-terminus activates into active ester in Marking 2, and the washing by PBS after activation in Marking 3. At that time the response value of the SPR falls back to baseline. The fixation of the bioprobe H_11_-OVA is shown in Marking 4, and the response value increases drastically. The fixed probe is monitored in real time. The response value no longer increases after about 45 minutes, meaning the bioprobe fixed on the biochip has been saturated. The fixation of the bioprobe is complete and is washed by PBS as shown in the Marking 5. At this time the response value of the SPR decreases slightly but is higher than the baseline in Marking 3, which suggests that the fixation effect is good. In Marking 6, the rest of the ester linkages are inactivated and confined with ethanolamine in order to avoid the formation of nonspecific adsorption. Marking 7 is the cleaning process by PBS to get rid of the substances that are not fixed. With all the operations above, the preparation of the biochip is complete and can be used for the detection of samples.

The antibodies against Coumaphos are diluted with PBS, standing for 5 minutes to ensure sufficient reaction after the antibody was mixed with samples of Coumaphos molecules. The concentration of the antibodies in the mixture is 10 mg/L, the concentrations of Coumaphos molecules are 0 µg/L, 50 µg/L, 100 µg/L, 300 µg/L, 500 µg/L, 1000 µg/L, 3000 µg/L and 5000 µg/L, the mixtures are added on the surface of the biochip. Then, the dynamic variation of the SPR is recorded. As Markings 8, 10, 12, 14, 16, 18, 20, 22 plot the dynamic curve of samples in Figure 2B, the concentrations of Coumaphos molecules are 5000 µg/L, 3000 µg/L, 1000 µg/L, 500 µg/L, 300 µg/L, 100 µg/L, 50 µg/L and 0 µg/L, respectively. Thus it can be seen when the concentrations of the samples decrease, the response value of SPR is increasing.

**Figure 3:**
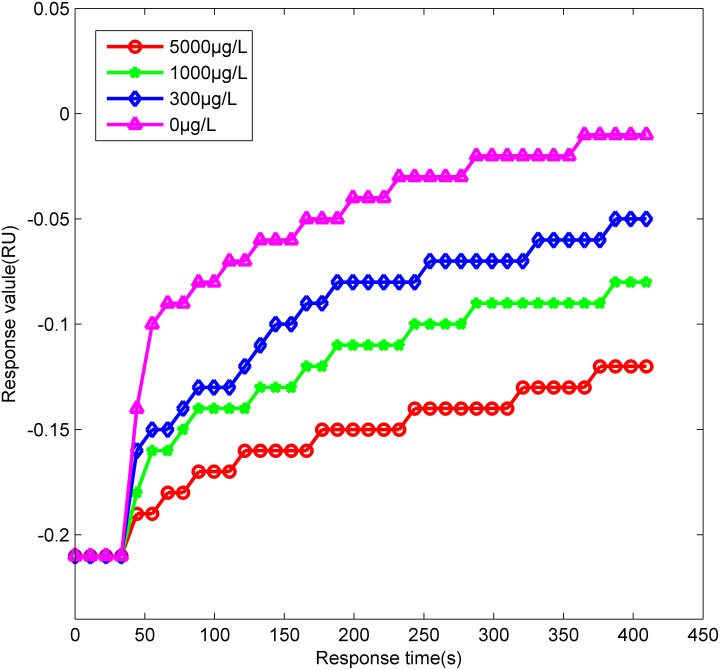
The dynamic curve of Coumaphos molecules detected by suppression method. The concentrations of samples are 0 µg/L, 300 µg/L, 1000 µg/L and 5000 µg/L.

There is a competitive relationship between the Coumaphos molecules in the mixture and the probe H_11_-OVA on the surface of the biochip, as both of them can be combined with the antibody in the mixture. The Coumaphos molecules suppress the combination between the antibody and the probe H_11_-OVA on the surface of the biochip. The antibody of Coumaphos is macromolecule, and Coumaphos is micromolecule. The molecular weight of the antibody is larger than Coumaphos, and the weight of H_11_-OVA-antibody is larger than Coumaphos-antibody. After the combination between the antibody and the probe H_11_-OVA on the surface of the biochip, the antigen-antibody conjugates on the surface of the biochip increase while the SPR response value also increases. The concentration of Coumaphos molecules is inversely proportional to the response value of SPR. If the concentration of Coumaphos molecules is low more antibodies of Coumaphos will combine with the probes on the surface of the biochip, and then the response value of SPR, namely the resonance angle, will become larger, as is shown in Fig. 2 and Fig. 3. Six minutes after the immune reaction, the SDS-HCl solution is used to elute the combination of the antigen and antibody. The PBS is used to wash the SDS-HCl after two minutes. The response value of SPR can be returned to the baseline, as is shown in Markings 9, 11, 13, 15, 17, 19, 21, 23 of Figure 2B.

Figure. 3 shows the dynamic curve of the partial samples, the concentrations of samples are 0 µg/L, 300 µg/L, 1000 µg/L and 5000 µg/L, respectively. With the concentration of Coumaphos molecules increasing, the response value of SPR declines.

In this case, the biochip with the fixation to the bioprobe can be used in the continuous detection of 80 groups of samples. The response value of SPR declines significantly and the sensitivity decreases after the detection of 80 groups of samples, signaling the bioprobe fixed on the surface of the biochip is damaged. The biochip regeneration can be realized after 0.1 mol/L HCl is added on the surface of the biochip. The sensor biochip can still be used after self-assembling and the fixation of bioprobe.

**Figure 4:**
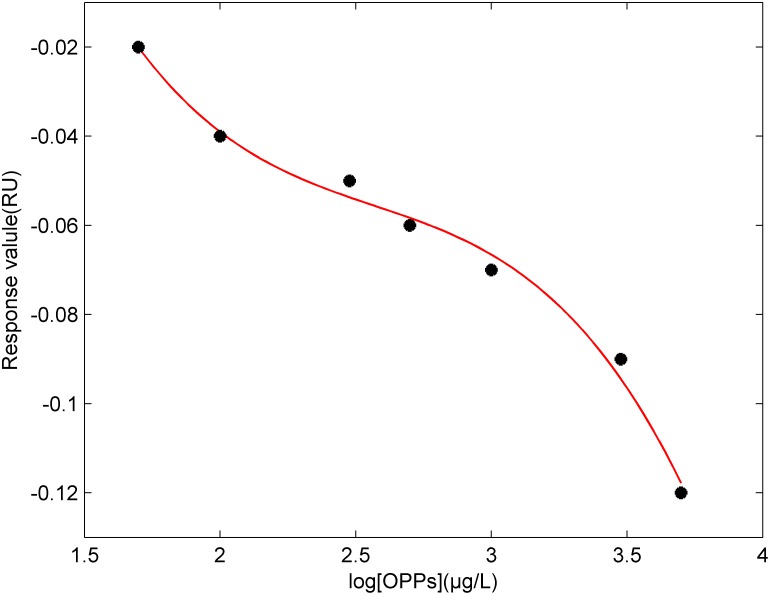
Standard curve of detecting Coumaphos molecules by suppression method. The y-coordinate represents the response value, and the x-coordinate is the concentration of Coumaphos. The curve appears approximately to be an inverted “S”. The value of IC_50_ is 1000 µg/L.

Figure 4 shows the standard curve of immunoreaction of Coumaphos molecules tested by suppression method with the SPR biochip. The y-coordinate represents the response value of the immune reaction at 5 minutes, and the x-coordinate is the concentration of Coumaphos. The curve appears approximately to be an inverted “S”. The standard deviation is less than 10% after the repeated detections of each concentration (3 times). The value of IC_50_ is 1000 µg/L. The detection limit is less than 25 µg/L. After detections of the SPR response value, the concentration of Coumaphos in the sample could be obtained by checking the standard curve. This method could contribute greatly to the quality control of fruits and vegetables and in-field real time detection.

To prepare different concentrations of Coumaphos standard solution, the concentrations of samples are 50 µg/L, 100 µg/L, 500 µg/L, 1000 µg/L, respectively, as the true value of samples. The content of Coumaphos molecules in Coumaphos standard solution is detected by using the SPR biochip in suppression method. And the estimated value of the concentrations of Coumaphos molecules can be calculated by the combination of the data detected by SPR detector and the standard curve shown in Fig. 4. The concentration of the Coumaphos standard is regarded as the real value and the data calculated by the standard curve is regarded as the estimated value. The real value compares with the estimated value, as is shown in Table 1. The absolute deviation of the real value compares and the estimated value is smaller, and so is the relative deviation. The SPR biochip method is effective, and the experimental method is feasible, and may successfully be used in food quality control and real-time detection.

**Table 1:** The analysis of the detection of Coumaphos molecules in Coumaphos standard solution by using the SPR biochip in suppression method.

Concentration(µg/L)	concentration measuredSPR detector (µg/L)				standarddeviation	absolutedeviation	relativedeviation
	estimatedvalue			averagevalue			
50	50.2	49.7	49.7	49.867	0.289	−0.133	0.58%
100	100.9	100.5	100.4	100.600	0.265	0.600	0.26%
500	501.2	502.1	504.4	502.567	1.650	2.567	0.33%
1000	998.5	1002.4	1004.5	1001.800	3.045	1.800	0.30%

The absolute deviation of the real value compares and the estimated value is smaller, and so is the relative deviation.

## Conclusions

Organophosphorus, specifically Coumaphos, has become one of the main pesticides for the prevention and control of plant diseases and insect pests. Excessive pesticide residues in fruits and vegetables, however, threaten the health of human beings and animals directly. As the inspection of food imports and exports becomes stricter, it is urgent and necessary to build a simple, effective and cheap detection method of pesticide residue for avoiding pesticide poisoning and protect the health of humans [37].

The detection of Coumaphos by using the biochip of SPR in suppression method mentioned in this paper, has a lot of advantages including inexpensive equipment, label-free detection, high accuracy, specificity and low cost. It also does not require a second antibody, chemical substances, such as fluorescence dye, and use unpurified antibody ascites. It can be used to detect pesticide residues in fruits and vegetables and quickly provide quantitative results. The equipment utilized is inexpensive, portable, environmentally friendly and easy to control. It ensures the achievement of real-time detection and rapid screening of large amount of samples making it ideal for usage in environments such as supermarkets, bazaars and factories.

In this paper, the adopted working concentration of antibody is 10 mg/L, if the working concentration of antibody is reduced, the detection limit of the sample of small Coumaphos molecules can also be reduced.
